# Exploration of miRNAs promoting eosinophilia and type 2 immunity

**DOI:** 10.3389/falgy.2026.1968077

**Published:** 2026-09-25

**Authors:** Hiba Sayedali, Jenny Calvén, Emma Boberg, Cecilia Lässer, Frida Dahlberg, Julie Weidner, Carina Malmhäll, Madeleine Rådinger, Kristina Johansson

**Affiliations:** Department of Internal Medicine and Clinical Nutrition, Krefting Research Centre, University of Gothenburg, Gothenburg, Sweden

**Keywords:** bone marrow, eosinophil (EOS), lung, miRNA, type 2 immunity

## Abstract

**Background:**

MicroRNAs (miRNAs) are evolutionary conserved, small non-coding RNAs that regulate gene expression and modulate cellular responses. In this study, we profiled miRNAs regulated by interleukin-33 (IL-33), a key inflammatory mediator of allergic responses and type 2 immunity that promotes bone marrow eosinophil development and subsequent airway recruitment.

**Method:**

C57BL/6 mice were exposed to recombinant IL-33 intranasally to induce airway eosinophilia. Cellular composition was determined by differential cell counts in bronchoalveolar lavage fluid and bone marrow. RNA profiling was performed using miRNA microarray, NanoString nCounter (miRNA and mRNA), qPCR (miRNA), and PrimeFlow—a method combining flow cytometry with single-cell miRNA detection.

**Result:**

IL-33 challenge induced bone marrow eosinophilopoiesis and a significant influx of eosinophils into the airways. IL-33 altered miRNA expression in the lungs, displaying a distinct profile compared to IL-33-responsive miRNAs in the bone marrow. miRNAs previously implicated in regulating eosinophil development (e.g., miR-223-3p and miR-21) and type 2 lymphocyte effector functions (e.g., miR-155-5p, miR-150-5p) were significantly altered. These changes were accompanied by gene expression alterations in mediators associated with eosinophil migration (e.g., *Il13ra1*, *Il4ra* and *Csf2rb*). miR-150-5p was downregulated in the bone marrow of IL-33-challenged mice, and further single-cell analysis via PrimeFlow identified an enrichment of miR-150-5p expression in ILC2s. Furthermore, miR-150-5p targets expressed in ILC2s were modulated by IL-33, which induced high IL-5 production in bone marrow ILC2s.

**Conclusion:**

We identify candidate miRNA programs involved in the progression of IL-33-dependent eosinophilic inflammation from bone marrow induction to lung recruitment.

## Introduction

Eosinophilic inflammation is a hallmark of type 2 immunity and a central immunopathological feature of allergic diseases, including asthma, allergic rhinitis, and atopic dermatitis (1). Upon activation, eosinophils act as potent effector cells that induce lung tissue damage through the release of an arsenal of cytotoxic and inflammatory mediators (1). Recent research has uncovered functional diversity within distinct eosinophil subsets, which has been associated with selective treatment responses in asthma (2, 3). Consequently, critical questions have emerged regarding how eosinophils are modulated, and whether distinct regulatory programs operate at the primary site of eosinophil development in the bone marrow compared to peripheral effector sites like the lung.

MicroRNAs (miRNAs) are gene-regulatory RNA molecules that display cell- and tissue-specific expression profiles. In asthma, robust and consistent changes in miRNA expression are detected in both airway biospecimens (4) and circulating blood samples (5, 6). These dysregulated miRNAs reflect both altered cellular composition and pathological disease states within specific cellular subsets (7). Indeed, several studies have leveraged alterations in miRNA expression to identify critical pathways driving disease pathology, including type 2 cytokine production (8), T cell polarization (8, 9), type 2 innate lymphoid cell (ILC2) activation (10, 11), and mucus hypersecretion (12)—all of which ultimately support and sustain airway eosinophilia (13, 14).

Interleukin-33 (IL-33) is a key player in type 2 immunity, which is supported by a large body of evidence including genome-wide association studies (GWAS) of allergic diseases (15). Released from the airway epithelium upon injury or stress, IL-33 orchestrates both innate and adaptive immune responses (16). It initiates a rapid cascade of events, including the immediate activation of ILC2s via the IL-33 receptor (ST2), leading to robust secretion of type 2 cytokines which drive eosinophilic inflammation (17). Additionally, IL-33 signaling enhances the activation of antigen-specific T helper 2 (Th2) cells (14). Importantly, the effects of IL-33 extend beyond the local airway mucosa; it also drives early progenitor development in the bone marrow by upregulating IL5R*α* on committed eosinophil progenitors (18), and induces IL-5^+^ ILC2s (19), thereby accelerating the maturation and subsequent systemic release of eosinophils (18).

In this study, we profiled the miRNAs regulated by IL-33 locally in the airways and distally in the bone marrow (Figure 1). We demonstrate candidate miRNA expression programs driven by IL-33 in the lungs versus the bone marrow reflecting tissue-specific processes that support peripheral immune cell activation and immune cell development during inflammation, respectively. Several miRNAs identified in our study are widely dysregulated in type 2 inflammatory diseases. A subset of these miRNAs have previously been directly implicated in regulating eosinophil differentiation and type 2 effector functions, providing essential cues that promote eosinophil development, survival, and peripheral migration. Ultimately, mapping this distinct transcriptional regulation by miRNAs in the bone marrow versus lungs may in future studies unveil specific pathways to dampen excessive bone marrow eosinophilopoiesis.

**Figure 1:**
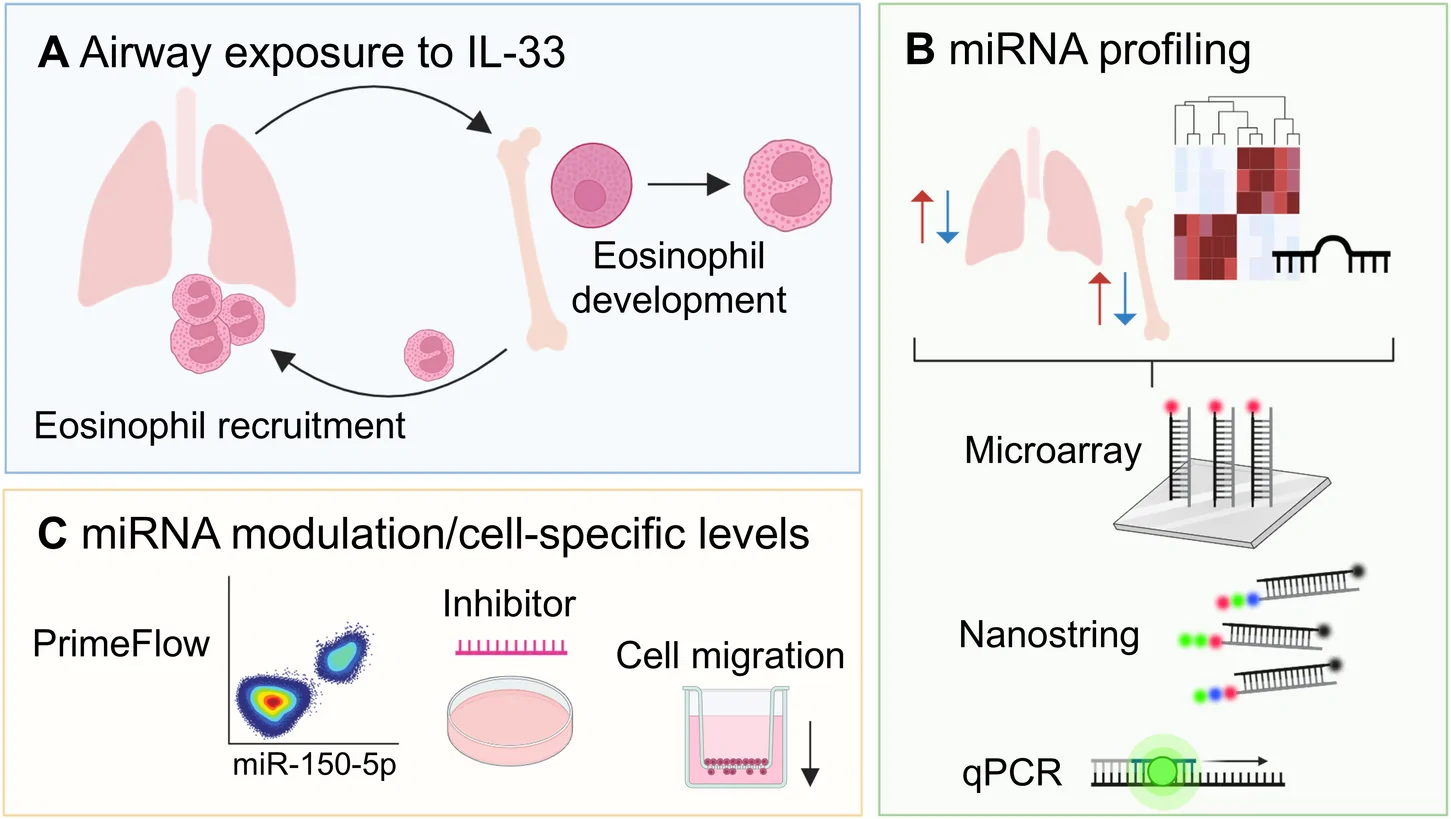
Graphical summary of the study. **(A)** Airway IL-33 challenge activates the development of eosinophils in the bone marrow which are recruited to the airways. **(B)** miRNA profiling was performed by miRNA microarray, Nanostring technology and qPCR analysis. **(C)** miRNA expression in cellular subsets was assessed by PrimeFlow analysis and *ex vivo* cultures examined IL-33-induced cell migration and miRNA modulation by inhibitor.

## Methods

### Mice

C57BL/6J wild type (WT) mice were purchased from Charles River (Germany), and miR-155 deficient mice were bred in-house. Sex- and age-matched mice were used in all experiments at 9–12 weeks of age. Mice were housed in pathogen-free conditions and given food and water *ad libitum.* Ethical permission for animal experiments was approved by the Gothenburg County Regional Ethical Committee.

### Induction and assessment of type 2 inflammation

Mice were exposed to 1 µg recombinant murine (rm)IL-33 (PeproTech) intranasally every other day for 5 days (Figure 2A). Control mice were given intranasal PBS and the mice were sacrificed 24 h after the final exposure. Bronchoalveolar lavage fluid (BALF) and bone marrow cells were collected. BALF was obtained by inflating the lungs with cold PBS (0.25 mL × 2) via a cannula inserted in the trachea. Bone marrow cells were collected by flushing the femur with 5 mL 2% FBS in PBS through a 100 µm cell strainer (CellTrics®, Sysmex). BALF and bone marrow cells were used to make cytospins (maximum 50,000 cells spun at 425 × g for 6 min). Samples were stained by Hemacolor® Rapid stain (Merck, Darmstadt, Germany) according to the manufacturer's protocol. 300 cells were morphologically assessed per cytospin using an Axioplan 2 microscope (Carl Zeiss).

**Figure 2:**
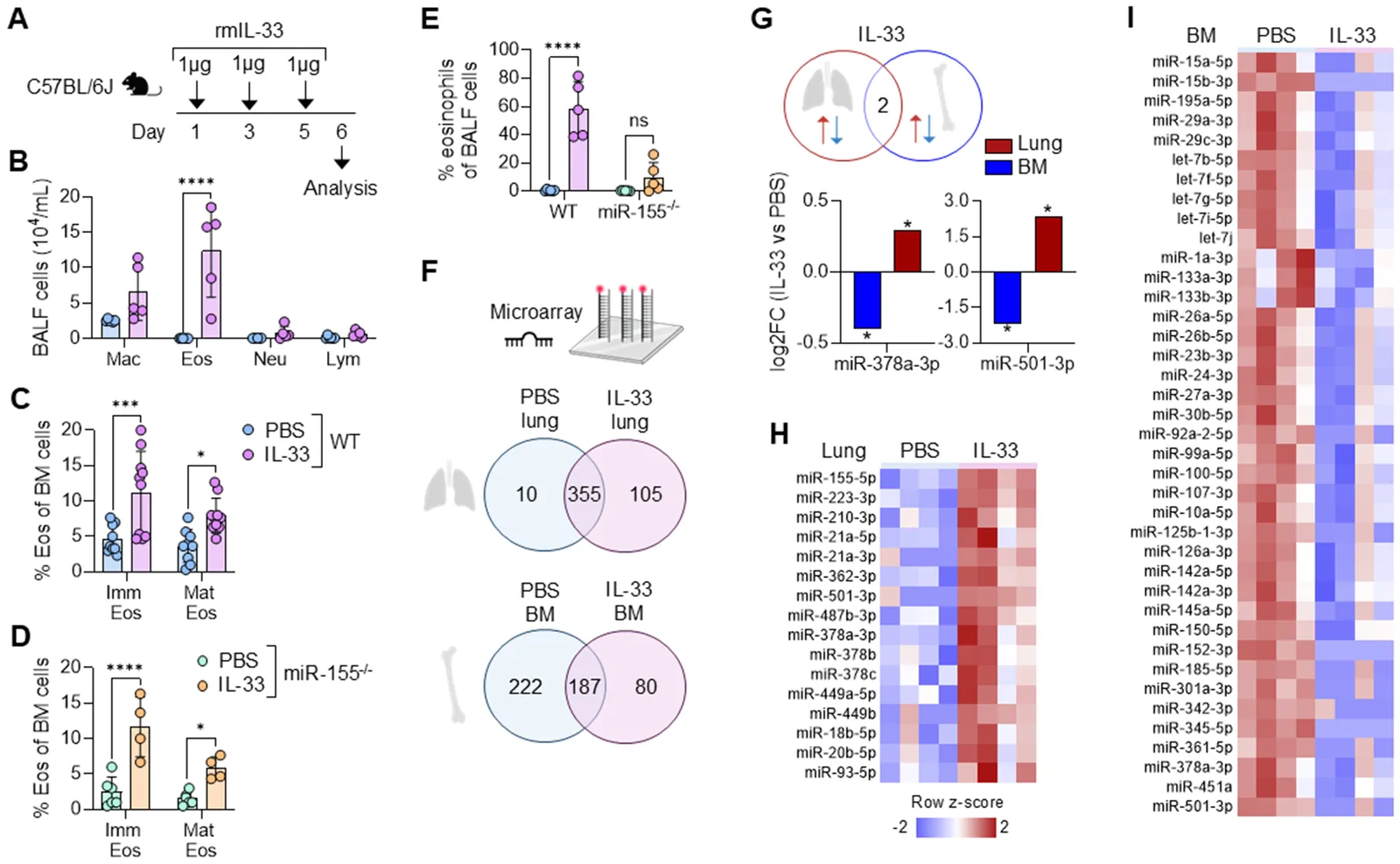
IL-33 induces eosinophilopoiesis in the bone marrow and is accompanied by tissue-specific alterations of miRNA expression. **(A)** Model of IL-33-mediated induction of type 2 airway inflammation. Cellular infiltration in bronchoalveolar lavage fluid (BALF; Macrophages, Mac; Eosinophils, Eos; Neutrophils, Neu; Lymphocytes, Lym) **(B)**, and frequency of immature (Imm Eos) and mature eosinophils (Mat Eos) in bone marrow (BM) of IL-33 exposed and PBS treated wildtype (WT) mice **(C)** and miR-155^−/−^ mice **(D)** mice. **(E)** Eosinophil induction shown by frequency of BALF cells in IL-33 exposed WT and miR-155^−/−^ mice. **(F)** Microarray analysis of lung tissue and BM of IL-33 exposed, and PBS control WT mice identified unique and shared miRNAs. **(G)** Two miRNAs (miR-378a-3p, miR-501-3p) were regulated by IL-33 in both lung and BM. Heatmap of miRNAs modulated by IL-33 (*P* < 0.05) in lung **(H)** and BM **(I)** of IL-33 and PBS WT mice. *N* = 4-9 mice/group. Two-way ANOVA with Šídák's multiple comparisons test in B-E and unpaired t test two-tailed in H-I. Bar graphs are shown with error bars demonstrating standard deviation. *p < 0.05, ***p < 0.001, and ****p < 0.0001 for significance.

In experiments where bone marrow cells were used for RNA analysis, femurs or tibias were flushed with RNA cell protect (Qiagen). Cells were centrifuged at 400xg for 5 min at 4 °C and pellets were resuspended in Qiazol (Qiagen) and stored at −80 °C until RNA extraction. The left lung lobe used for RNA analysis was collected in RNAlater (Qiagen) and stored at −80 °C until RNA extraction.

### RNA isolation

Total RNA from bone marrow and lung tissue was isolated using Qiagen miRNeasy mini or micro kits according to the manufacturer's protocol, including tissue homogenization by Qiashredder columns (Qiagen). RNA concentration and integrity was measured on an Agilent 2100 bioanalyzer running Agilent RNA 6000 pico chips (Agilent Technologies), a NanoDrop^TM^ spectrophotometer or a DeNovix spectrophotometer.

### miRNA profiling by microarray and NanoString platforms

For microarray analysis, bone marrow and lung RNA samples (*n* = 4/group) were normalized to 125 ng/µL and 2.2 µL sample was mixed with miRNA spike-in (catalog no TRT XR304, Toray) and further labelled, hybridized and washed according to the instruction manual from Toray (H-M-R miRNA protocol 4-Plex V3) using Toray miRNA Labeling kit (TRT-XE211) and Mouse miRNA Oligo chip 4plex, based on miRBase 21 (TRT-XR520, Toray). The analysis was performed by TATAA Biocenter, Gothenburg, Sweden. The intensity of each miRNA was determined using a 3D-Gene Scanner 3000 (Toray industries Inc., Tokyo, Japan). All procedures were carried out according to the manufacturer's instructions. A miRNA was determined to be present if expressed in three out of four samples within its own group (*n* = 4) and differently expressed if the fold change was > 2 and *p*-value < 0.05 (student's *t*-test) when comparing the IL-33-challenged group with the PBS control group.

For NanoString analysis, bone marrow RNA samples (*n* = 6/group) were processed for miRNA or mRNA analysis (Mouse v1.5 miRNA or Immunology panels) using NanoString nCounter® Analysis System according to the manufactureŕs protocol. The analysis was performed by the SpatialOmics core facility at Lund University, Lund, Sweden. Briefly, 100 ng of total RNA was used for tag annealing, ligation and hybridization to reporter and capture probes in a thermal cycler (20 h, 65 °C). The NanoCounter PrepStation was used for washing and cartridge immobilization. The cartridge was scanned 555 fields of view using a Digital Analyzer and nSolver 4.0 software was used for data analysis. Samples were normalized to the positive endogenous controls and to the top 100 expressed miRNAs or mRNAs in the panels.

### miRNA profiling by qPCR

100 ng total RNA in 2 µL was used to make cDNA by reverse transcription reaction using the miRcury LNA RT kit (Qiagen). As a control, UniSp6 RNA Spike-in was added into each reaction in the cDNA synthesis step. qPCR analysis was performed on a Bio-Rad CFX96 real time PCR detection system. Reactions were set up using miRCURY LNA primers and miRCury SYBR green (Qiagen) according to the manufacturer's instructions. RNU5 g was used as reference RNA for expression studies of miRNAs in *in vivo* experiments and miR-103-3p was used as reference miRNAs in *in vitro* experiments. Pre-designed miRCury LNA primers (Qiagen) for mmu-miR-150-5p was used.

### miRNA profiling by PrimeFlow

Bone marrow cells were stained with antibodies against surface markers to detect ILC2s and CD4^+^ T cells (Lineage cocktail-FITC, CD19-FITC, NK1.1-FITC, CD11c-FITC, FcERI-FITC, ST2-PE, CD45-PerCP, CD4-APC-H7, CD127-BV421) and eosinophils (Siglec-F-AF488, CD34-PE, CD45-PerCP, CD125-RB780, CCR3-BV421) and LIVE/DEAD™ Fixable Aqua Dead Cell Stain Kit for 405 nm excitation was used to distinguish live cells. For miRNA detection, we used AF647-miR-150-5p-probe Type-1 and AF488-ACT-B was used as control according to the manufacturer's protocol (ThermoFisher Scientific). The flow cytometric analysis was carried out on a BD FACSVerse and data was analyzed using FlowJo software.

### Migration assay

1 × 10^6^ bone marrow cells were resuspended in 100 µL culture medium (RPMI-1640 supplemented with 5% FBS) and added to the apical (upper) chamber of Corning® Transwell® 24 well plate with 3.0 μm pore size. 600 µL of culture medium was added to the basolateral (lower) chamber. Recombinant IL-33 (100 ng/mL), CCL24/Eotaxin-2 (250 ng/mL) or combination (IL-33+ CCL24, both obtained from ThermoFisher Scientific) were added to the basolateral chamber. Cells were incubated at 37 °C, 5% CO₂ for 6 h. Non-migrated (apical chamber) and migrated cells (in basolateral chamber and trypsinized from filter) were collected and counted. Eosinophils were identified by differential cell count of cytospin preparations.

### Modulation by miRNA inhibitors

In experiments using a miR-150-5p inhibitor, 2 ×  10^6^ bone marrow cells/mL were added to 24-well plates in complete cell culture medium (RPMI-1640 supplemented with 10% FBS, 2 mM L-glutamine, 100 U/mL penicillin, 100 µg/mL streptomycin, and 1 mM sodium pyruvate). Medium control, non-targeting control power inhibitor or miR-150-5p power inhibitor (Qiagen) were added to the cells at 100 nM concentration in 2 µL. At the same time, 100 ng/mL rmIL-33 (Peprotech) was added to the cells and incubated for 48 h. 3 h before harvest (for analysis of intracellular cytokines), cells were restimulated with IL-33 (100 ng/mL) and Monensin (BD GolgiStop^TM^) was added at a concentration of 4 µL/6 mL.

### Intracellular flow cytometry

For cytokine analysis, cells were suspended in 50 µL 2% mouse serum with GolgiStop. Surface makers were stained using antibodies (Lineage cocktail-FITC, CD19-FITC, NK1.1-FITC, CD11c-FITC, FcERI-FITC, CD127-PE, CD45-PerCP, ST2-APC) for 30 min at 4 °C (dark), then washed twice and fixed with BD cytofix and incubated for 15 min at RT (dark). Cells were washed with PBS and resuspended in 0.1% saponin buffer (SAP) in HBSS then centrifuged. Intracellular antibodies (IL-10-APC-Cy7, IL-5-BV421) were incubated for 40 min at RT (dark), then washed with SAP buffer. Cells were washed again with 1% FBS in PBS and resuspended in 250 µL 1% FBS in PBS for flow cytometric analysis on a BD FACSVerse instrument. Analysis was done using FlowJo software.

### Statistical analysis

Microsoft Excel and GraphPad Prism Software were used for data analysis. *Z* score was calculated from mean and standard deviation. *p < 0.05, **p < 0.01, ***p < 0.001, and ****p < 0.0001 for significance. ANOVA was used as statistical test of multiple groups, with appropriate *post hoc* testing as stated in the text. All data was assumed to be normally distributed.

## Results

### Airway exposure to IL-33 induces bone marrow eosinophil maturation and airway migration

To establish a model of eosinophilic inflammation, we subjected mice to repeated stimulation with IL-33. IL-33-challenged mice exhibited high numbers of eosinophils in the BALF, as determined by differential cell count analysis (Figure 2B). A corresponding increase in both immature and mature eosinophils was identified in the bone marrow (Figure 2C), indicating that the airway challenge model activated type 2 immune responses by engaging the bone marrow compartment. Next, we evaluated the role of miRNA regulation in this axis using mice with a global deficiency in miR-155. Following IL-33 challenge, miR-155 deficient mice demonstrated similar induction of bone marrow eosinophils (Figure 2D) to that seen in WT mice (Figure 2C). However, airway eosinophilia was severely attenuated in miR-155 deficient mice (Figure 2E), highlighting tissue-specific requirements for miR-155 during IL-33-driven eosinophilic inflammation.

### IL-33 stimulation alters global miRNA expression profiles in the lungs and bone marrow

Given these tissue-specific differences, we next sought to comprehensively map the miRNA expression profiles in IL-33-driven inflammation. We analyzed miRNA levels in the lung and bone marrow of IL-33-challenged mice and PBS controls via microarray, detecting 470 miRNAs in the lungs (Figure 2F, top), and 489 miRNAs in the bone marrow (Figure 2F, bottom). In the lungs, 76% of the detected miRNAs (355) were shared between IL-33 mice and PBS controls, whereas only 38% of miRNAs (187) were shared between the treatment groups in the bone marrow. Notably, only two miRNAs were significantly altered by IL-33 in both compartments. miR-378a-3p and miR-501-3p displayed significantly upregulated expression in the lungs after IL-33 challenge but were downregulated in the bone marrow of the same mice (Figure 2G). Furthermore, miR-378a-3p expression levels, but not miR-501-3p, demonstrated a significant negative correlation between the lungs and bone marrow (Supplementary Figure S1).

In the lungs, several miRNAs previously implicated in type 2 immunity were significantly upregulated by IL-33 (Figure 2H), including miR-155-5p (10, 20), miR-223-3p (21, 22), miR-21 (23), miR-18b-5p/miR-20b-5p (8, 11)), miR-210 (24) and miR-449a/b (25). Conversely, a larger subset of miRNAs was significantly downregulated in the bone marrow following IL-33 challenge (Figure 2I). Bone marrow miRNAs included key regulators of lymphoid effector cell differentiation, such as miR-29a/c-3p (26), the miR-23∼27∼24 cluster (9, 27), and miR-15a/b-5p (28). Together, these distinct miRNA profiles reflect a coordinated response in both the lungs and bone marrow during IL-33-driven immune activation.

### The baseline bone marrow miRNA landscape remains stable following airway IL-33 exposure

To validate and further characterize bone marrow miRNA expression, we utilized an amplification-free nCounter miRNA expression panel by NanoString (covering nearly 600 miRNAs) to perform direct quantification of individual miRNA molecules. We first analyzed the most highly expressed miRNAs in the bone marrow of PBS control mice (Figure 3A, top 50 miRNAs). Notably, miR-223-3p which regulates eosinophil development and function (21, 22) was identified as the second most abundant miRNA in this compartment. We next evaluated miRNA abundance in the bone marrow following IL-33-induced airway inflammation. The profile of the most highly expressed miRNAs in the IL-33-challenged bone marrow demonstrated striking similarity to that of the PBS controls (Figure 3A, heatmap).

**Figure 3:**
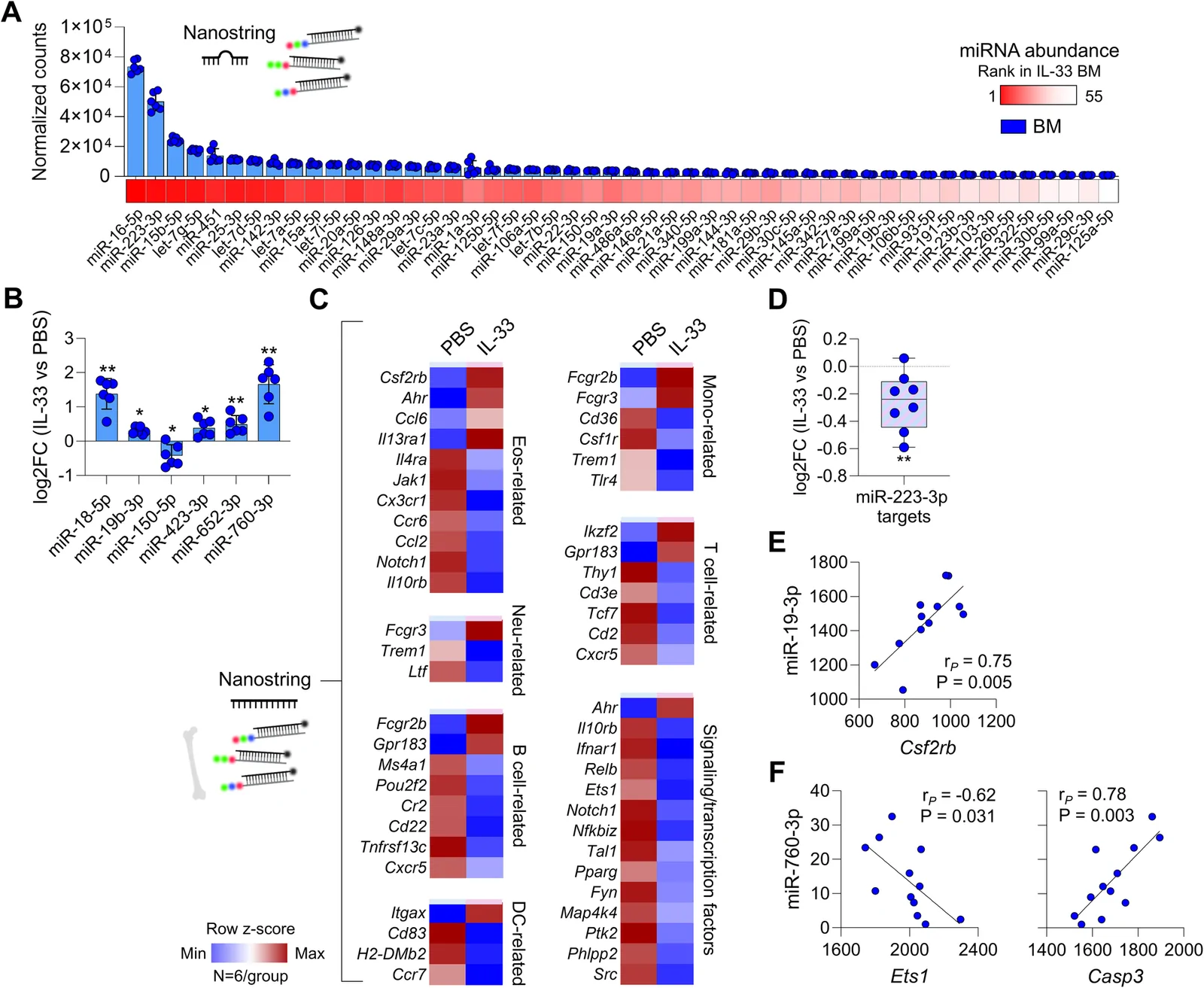
Absolute quantification of miRNAs in bone marrow reveals minor effect on relative abundance upon IL-33 exposure, but widespread changes in gene expression levels. **(A)** Bar graph demonstrating miRNA abundance in bone marrow (BM) of PBS control wildtype mice by Nanostring technology, and IL-33 exposed wildtype mice visualized in heatmap below (demonstrating rank from most highly expressed; 1). Significantly altered miRNAs **(B)** and mRNAs **(C)** in BM of IL-33 challenged wildtype mice compared to PBS control by Nanostring technology. **(D)** Modulation of miR-223-3p target genes in BM in response to IL-33. miR-19b-3p **(E)** and miR-760-3p **(F)** levels in BM shown as normalized counts versus expression levels of predicted target genes (normalized counts) assessed by Nanostring technology. *N* = 6 mice/group. Unpaired t test two-tailed in B-D, and *r_P_*, Pearson correlation coefficient in E-F. Bar graphs are shown with error bars demonstrating standard deviation. *p < 0.05, and **p < 0.01 for significance.

To validate these findings across platforms, we performed a parallel abundance analysis using our microarray dataset (Supplementary Figure S2A). Despite the inherent methodological differences between the two technologies, we observed a substantial overlap between the top miRNAs identified by microarray and NanoString profiling (Supplementary Figure S2B). In agreement with the NanoString data, miR-223-3p was independently identified by microarray as the second most highly expressed miRNA in the bone marrow (Supplementary Figure S2B). Overall, 31 out of the top 50 most abundant miRNAs were consistently identified by both platforms (Supplementary Figure S2B). Finally, an assessment of coverage overlap revealed that 61 miRNAs included in the nCounter panel were not represented on the microarray chip (Supplementary Figure S2C).

### Cross-platform profiling identifies a restricted subset of IL-33-responsive bone marrow miRNAs

Having cross-validated overall miRNA abundance across both platforms, we next evaluated which specific bone marrow miRNAs were significantly modulated by IL-33-induced airway inflammation using NanoString. Differential expression analysis revealed that only six miRNAs were significantly altered in the bone marrow following IL-33 challenge (Figure 3B). Specifically, miR-18-5p, miR-19b-3p, miR-423-3p, miR-652-3p, and miR-760-3p were increased, while miR-150-5p expression was decreased (Figure 3B). Notably, the reduction in miR-150-5p was the only consistent hit between the differentially expressed miRNAs identified by both the NanoString and microarray analyses. Furthermore, miR-378a-3p which displayed a negative lung-to-bone-marrow correlation in our microarray data was not included in the nCounter miRNA expression panel.

### Airway IL-33 exposure modulates the bone marrow transcriptomic landscape and type 2 cytokine receptor expression

To further characterize the downstream cellular responses in the bone marrow following IL-33 challenge, we performed targeted gene expression profiling using the nCounter Immunology panel (∼600 mRNAs by NanoString detection). We identified 66 differentially expressed genes in the bone marrow of IL-33-challenged mice compared to PBS controls (Figure 3C; Supplementary Figure S3). These transcriptomic alterations included genes associated with different immune cell subsets. Of particular interest, *Csf2rb* (GM-CSF receptor subunit beta), a critical component of the receptor complex for IL-5 and GM-CSF, was significantly decreased in the bone marrow of IL-33-challenged mice (Figure 3C). Additionally, expression of the type 2 cytokine receptors *Il13ra1* and *Il4ra* was significantly modulated after IL-33 challenge, with *Il13ra1* expression increasing and *Il4ra* expression decreasing (Figure 3C).

To link these transcriptomic changes to miRNA regulation, we analyzed evolutionary conserved miRNA targets within our gene expression dataset using TargetScan Mouse 8.0. This in silico analysis demonstrated a downregulation of predicted miR-223-3p target genes in the bone marrow of IL-33-challenged mice (Figure 3D). Furthermore, expression levels for a subset of the differentially expressed miRNAs identified by NanoString profiling, specifically miR-19b-3p and miR-760-3p, correlated significantly with the expression of their predicted target genes, including *Csf2rb*, *Ets1*, and *Casp3* (Figures 3E,F).

Taken together, these results demonstrate that airway IL-33 exposure alters the bone marrow transcriptomic landscape, specifically modulating key receptors involved in eosinophil biology.

### Bone marrow miR-150-5p expression is modulated by IL-33 and enriched in distinct lymphoid subsets

Given that miR-150-5p was the only consistently altered miRNA across both profiling platforms, we sought to further characterize its expression in the bone marrow. qPCR validation confirmed a significant downregulation of miR-150-5p expression in the bone marrow of IL-33-challenged mice compared to PBS controls (Figure 4A). To investigate cell-specific distribution of miR-150-5p, we utilized PrimeFlow RNA assays combined with flow cytometry to analyze expression within distinct bone marrow immune cell subsets (Figure 4B). Using a miR-150-5p-specific probe, we evaluated ILC2s, CD4^+^ T cells, mature eosinophils and developing eosinophil progenitors. The highest expression levels of miR-150-5p were detected in ILC2s and CD4^+^ T cells, with substantially lower levels observed in both eosinophil populations (Figure 4C). Quantification of probe-positive cells further revealed a significantly higher frequency of miR-150-5p^+^ cells among ILC2s compared to CD4^+^ T cells (Figure 4D). Consistent with our profiling datasets, PrimeFlow analysis of total bone marrow showed a significant reduction in overall miR-150-5p expression following IL-33 challenge (Figure 4E, left). However, cell-specific analysis demonstrated that miR-150-5p levels within ILC2s (Figure 4E, middle) and CD4^+^ T cells (Figure 4E, right) remained unchanged after IL-33 stimulation.

**Figure 4:**
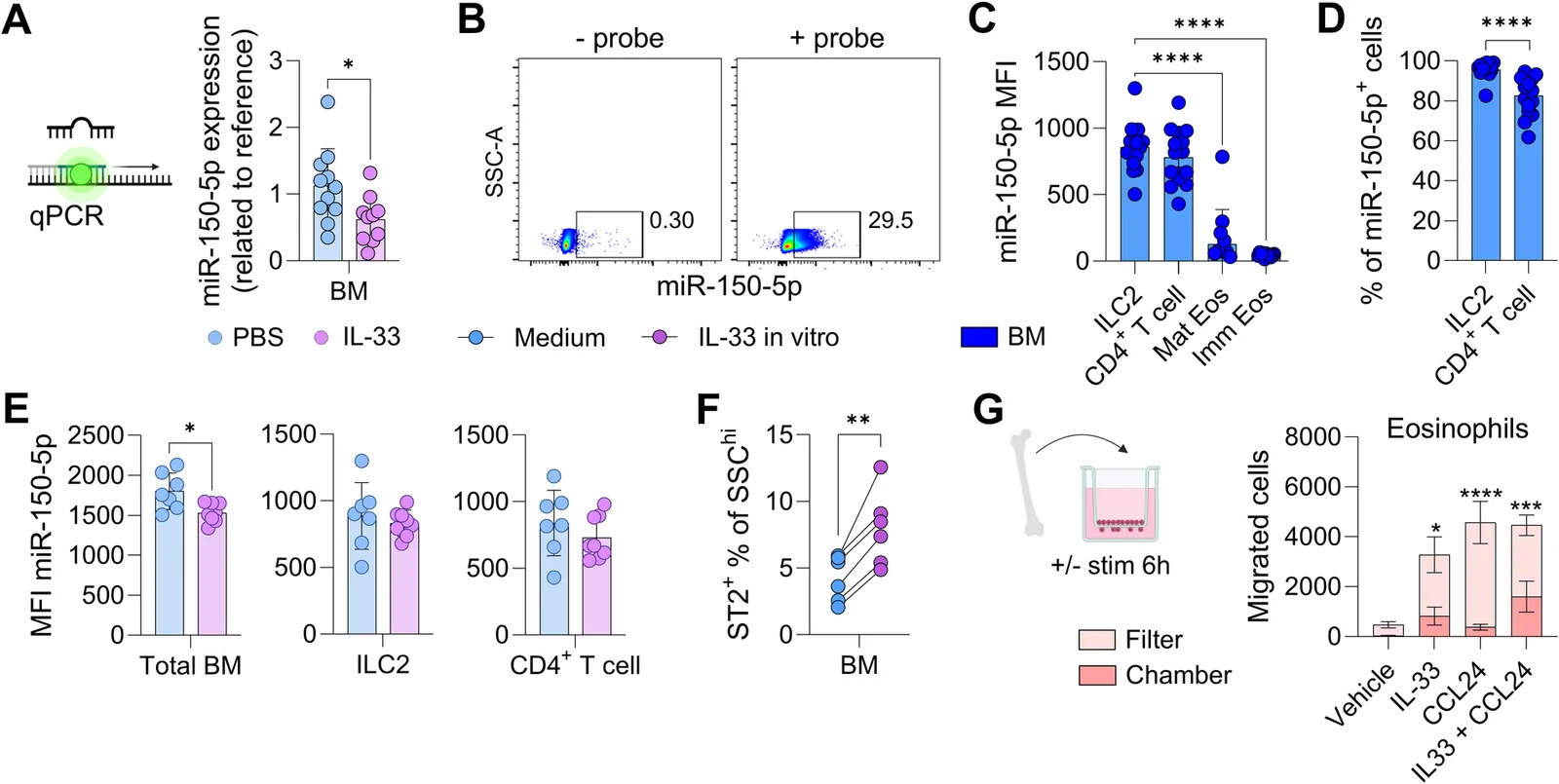
IL-33 alters miR-150-5p levels in the bone marrow and is differentially expressed in distinct immune cell subsets. **(A)** qPCR analysis of miR-150-5p levels in bone marrow (BM) of IL-33 exposed mice and PBS controls. PrimeFlow analysis demonstrating miR-150-5p expression using fluorescent probes **(B)**, as mean fluorescent intensity (MFI) **(C)** and frequency of miR-150-5p positive cells **(D)** in distinct immune cells in the bone marrow. **(E)** miR-150-5p expression by MFI in total BM cells (left), ILC2s (middle), and CD4^+^ T cells (right) of IL-33 exposed and PBS control mice, assessed by PrimeFlow analysis. **(F)** IL-33 receptor (ST2) positive SSC^hi^ BM cells with and without IL-33 stimulation (24 h). **(G)** Migration assay of BM cells with IL-33, CCL24 or the combination. *N* = 6-15 mice/group. Unpaired t test two-tailed in A, D-E, and ordinary ANOVA with Dunnett's multiple comparison test C and G. Bar graphs are shown with error bars demonstrating standard deviation in all except G which is showing standard error of the mean. *p < 0.05, **p < 0.01, ***p < 0.001, and ****p < 0.0001 for significance.

Because the reduction in overall bone marrow miR-150-5p coincided with the expansion of eosinophils (which exhibit low baseline miR-150-5p expression), we next examined the direct responsiveness of bone marrow eosinophils to IL-33. Flow cytometric analysis of SSC^hi^ eosinophils revealed a significant upregulation of the IL-33 receptor ST2 in IL-33-challenged mice compared to PBS controls (Figure 4F). To evaluate functional responsiveness, we performed an *in vitro* migration assay. Bone marrow eosinophils demonstrated significant migration in response to either IL-33 or CCL24/Eotaxin-2 alone, with the migration being further potentiated by combined IL-33 and CCL24 stimulation (Figure 4G). In summary, IL-33 acts directly on bone marrow eosinophils to upregulate ST2 and promote migration. However, our findings indicate that while the total bone marrow miR-150-5p level is affected by IL-33, its expression is primarily enriched in lymphoid effector subsets as compared to eosinophils.

### IL-33 primes bone marrow ILC2 functionality and drives distinct miR-150-5p target gene signatures

To evaluate the functional priming of bone marrow ILC2s, we stimulated bone marrow cells *ex vivo* (isolated from airway IL-33-challenged or PBS control mice) with IL-33 to assess IL-5 induction (Figure 5A). Bone marrow cells derived from airway IL-33-challenged mice generated a significantly higher number of IL-5^+^ ILC2s upon *ex vivo* re-stimulation compared to cells from PBS controls (Figure 5B). This increased functional responsiveness coincided with a significant upregulation of ST2 expression on ILC2s from the IL-33-challenged group (Figure 5C).

**Figure 5:**
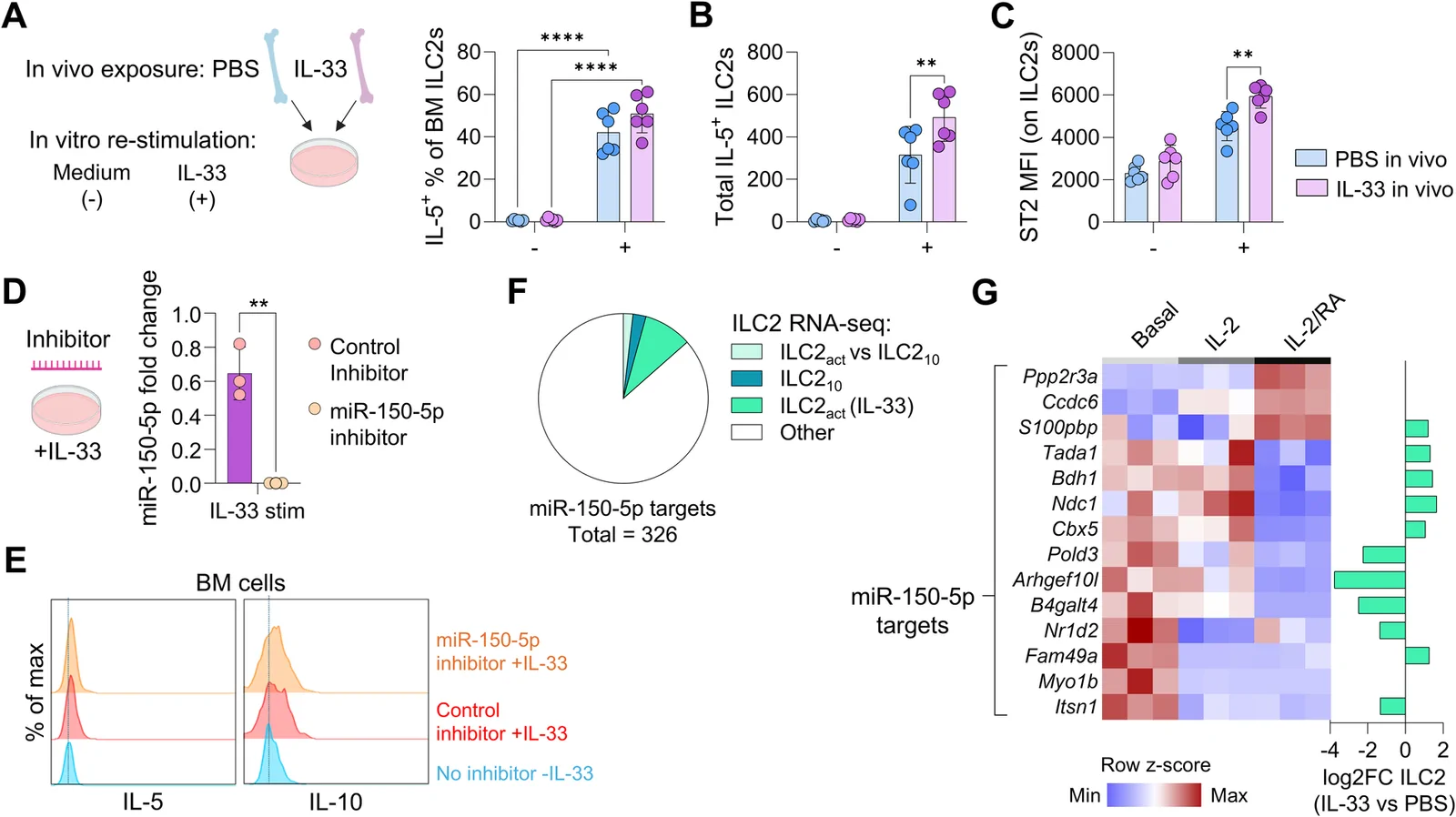
miR-150-5p target genes are modulated in ILC2s following activation. Frequency of **(A)** and total **(B)** IL-5^+^ ILC2s in bone marrow (BM) from IL-33 exposed or PBS control mice following *in vitro* IL-33 re-stimulation. **(C)** Mean fluorescent intensity (MFI) of the IL-33 receptor ST2 on ILC2s in the same mice. **(D)** qPCR analysis of miR-150-5p expression in bone marrow (BM) cultures stimulated with IL-33 and incubated with a miR-150-5p-specific inhibitor or non-targeting control inhibitor. **(E)** Cytokine production by BM cells with or without miR-150-5p inhibitor were assessed by intracellular flow cytometry. Pie diagram of all conserved TargetScan-predicted miR-150-5p target genes altered upon ILC2 activation **(F)** (29), and their expression in ILC2s cultured *in vitro* **(G)** (29). *N* = 3-6 mice/group. Two-way ANOVA with Šídák's multiple comparisons test in A-C and unpaired t test two-tailed in D. Bar graphs are shown with error bars demonstrating standard deviation. **p < 0.01, and ****p < 0.0001 for significance.

Next, we investigated whether synthetic oligonucleotides could modulate endogenous miR-150-5p activity within these bone marrow cultures using specific inhibitors. Bone marrow cultures were incubated with a miR-150-5p inhibitor or a non-targeting control inhibitor in the presence of IL-33 for 48 h. qPCR confirmed that miR-150-5p levels were significantly decreased in cultures treated with the specific inhibitor (Figure 5D). Given that miR-150-5p has been implicated as a negative regulator of ILC2 function (29), we analyzed IL-5 and IL-10 production in these cultures. However, cytokine levels remained comparable between the specific inhibitor and non-targeting control groups following IL-33 stimulation (Figure 5E).

To identify potential ILC2-intrinsic roles for this miRNA, we analyzed a dataset of 326 evolutionary conserved, TargetScan-predicted miR-150-5p targets against RNA-seq data from isolated ILC2s (30). Among these predicted targets, 44 genes were significantly modulated in activated ILC2s (Figure 5F). These target genes were differentially expressed in IL-33-activated ILC2s, with a distinct subset being altered in alternatively activated, regulatory ILC2s characterized by IL-10 production. Finally, we mapped these miR-150-5p target genes in ILC2s stimulated *in vitro* with IL-2 alone or in combination with retinoic acid to drive the regulatory phenotype (30), revealing consistent gene expression alterations across both *in vitro* and *in vivo* ILC2 activation models (Figure 5G). In summary, these data demonstrate that airway IL-33 exposure functionally primes bone marrow ILC2s and that IL-33-modulated genes in ILC2s includes conserved miR-150-5p target gene networks.

## Discussion

The current study underscores the importance of studying eosinophils at its source; the bone marrow. Our systemic profiling revealed that airway IL-33 challenge modulates miRNA expression both locally in the lungs and distally in the bone marrow compartment. Interestingly, these tissue environments presented highly distinct molecular features. For instance, miR-155-5p was upregulated exclusively in the lungs, while undetectable in the bone marrow. This compartmentalization may explain why global miR-155-5p deficiency selectively impairs airway ILC2 expansion and abolishes lung inflammation without disrupting induction of bone marrow eosinophilia (10, 20). It opens the possibility that airway ILC2s require miR-155-5p signaling, while bone marrow ILC2 populations function independently of it.

miR-378a-3p was uniquely altered in both tissues, demonstrating an inverse correlation between the lung (upregulated) and bone marrow (downregulated). Given its suggested role in regulating IL-33 expression (31), eosinophil differentiation (32), and its clinical association with poor bronchodilator responses in pediatric asthma (33), it is possible that miR-378a-3p is involved in different processes during eosinophilic inflammation.

Discrepancies between microarray and NanoString platforms are well-documented and are likely due to fundamental differences in underlying detection chemistries. NanoString nCounter utilizes a ligation-based tag extension system that is combined with single-molecule counting, providing higher sensitivity for detecting miRNAs at low-abundance. Microarrays, on the other hand, measure relative fluorescence intensity that rely on probe-based hybridization which can be constrained by variations in labeling efficiency and background noise (34). This method is inherently susceptible to cross-hybridization and signal saturation, narrowing its dynamic range and increasing false-positive rates compared to amplification-free direct digital quantification. This technical limitation partly explains why our microarray analysis detected a substantially higher number of differentially expressed miRNAs than the NanoString nCounter panel. Importantly, however, the relative baseline abundance rankings of top-expressed miRNAs largely overlapped between both technologies. To minimize false positives, we applied strict filtering (requiring detection in at least three out of four biological replicates), though requiring absolute presence across all samples could tighten future screening even further.

Targeted mRNA profiling of the bone marrow revealed IL-33-dependent modulation of genes indicating active eosinophil maturation and migration, such as *Csf2rb* and *Il4ra.* Because IL-5 and GM-CSF strictly regulate eosinophil survival and development (1), the upregulation of *Csf2rb* transcript levels may reflect increased eosinophil production which differential cell count analysis of IL-33 challenged mice confirmed. At the same time, *Il4ra* expression is known to prime eosinophils for enhanced chemotaxis toward eotaxins (35). Coordinated changes are further supported by our TargetScan analysis, which demonstrated an enrichment of downregulated miR-223-3p targets and correlation between specific modulated miRNAs (miR-19b-3p, miR-760-3p) and homeostatic genes (*Csf2rb*, *Ets1*, and *Casp3*).

Because miR-150-5p was the sole candidate consistently downregulated across microarrays, NanoString, and qPCR, we further assessed its cell-specific expression via PrimeFlow. Our data show that miR-150-5p is enriched within lymphoid lineages (ILC2s and CD4^+^ T cells) but expressed at low baseline levels in myeloid cells like eosinophils. While total bone marrow miR-150-5p levels decreased after IL-33 challenge, ILC2- and CD4^+^ T cell-specific levels did not exhibit a significant reduction. This contrast with a previous study by Singh et al. which reported robust miR-150-5p downregulation by IL-33 in primary ILC2s (11), may be due to experimental kinetics or site-specific miRNA changes. Singh et al. evaluated cultured ILC2s from lungs after 10 days of continuous *in vitro* IL-33 stimulation, whereas our model captured miR-150-5p expression *ex vivo* in the bone marrow following a 5-day IL-33 *in vivo* protocol. Furthermore, our *ex vivo* oligonucleotide experiments demonstrated that while a synthetic inhibitor successfully knocked down miR-150-5p, it did not alter IL-5 or IL-10 production. Nonetheless, our RNA-seq analysis confirmed that 44 conserved miR-150-5p target genes are actively modulated during both inflammatory (IL-33-driven) and regulatory (IL-10-producing) ILC2 activation states (30).

In summary, airway IL-33 exposure orchestrates a systemic response that rapidly remodels the bone marrow microenvironment. This response is driven by specific transcriptomic changes and tissue-specific miRNA networks that promote eosinophil maturation and peripheral recruitment. Dissecting these candidate pathways further may generate new insights into the molecular underpinnings of persistent eosinophilia.

### Clinical perspectives

Severe T2-high asthma is characterized by persistent airway eosinophilia, which correlates with disease severity and exacerbations (36, 37). Despite the clinical availability of biologics targeting IL-5 or IL-4/IL-13 pathways, a significant subset of patients exhibits residual tissue eosinophilia or paradoxical hypereosinophilia (38, 39). Emerging evidence suggests this resistance may be driven by functional eosinophil heterogeneity and selective IL-5-independent subpopulations (2, 40). Identifying miRNAs that control specific cellular trajectories during type 2 inflammation may lead to development of novel therapeutic strategies. Rather than systemically depleting the entire eosinophil pool, modulating miRNAs specific to the local microenvironment could selectively restrict bone marrow egress or reprogram mature cells into a less inflammatory state by changing their degranulation or activation threshold. Although preclinical studies in mice using miRNA antagomir administration have shown great promise by dampening airway inflammation and improving clinical features of disease such as airway hyperresponsiveness and mucus accumulation (25), technical and biological challenges still remain. For instance, delivery of miRNA-directed therapeutics needs to ensure efficient and safe targeting while being evaluated for long-term effects (41). An important focus area for assessing therapeutic potential of miRNAs in future studies includes use of miRNA reporter systems to carefully map miRNA activity in target cells during homeostatic and inflammatory conditions.

## Data Availability

The original contributions presented in the study are included in the article/Supplementary Material, further inquiries can be directed to the corresponding author
