# Supplementary material for: Exploration of miRNAs promoting eosinophilia and type 2 immunity
Source: Front Allergy. 2026 Sep 25;7:1968077. doi: 10.3389/falgy.2026.1968077 (PMC13649434; doi:10.3389/falgy.2026.1968077)
Supplement: Supplementary file 1 [file Datasheet1.pdf]

Exploration of miRNAs promoting eosinophilia and type 2 immunity

Hiba Sayedali[1], Jenny Calvén[1], Emma Boberg[1], Cecilia Lässer[1], Frida Dahlgren[1], Julie Weidner[1], Carina Malmhäll[1], Madeleine Rådinger[1] and Kristina Johanssson[1,*]

[1]*Krefting Research Centre, Department of Internal Medicine and Clinical Nutrition, University of Gothenburg, Gothenburg, Sweden.*

Supplementary figure legends

Supplementary Figure S1. Correlation analysis of miR-378a-3p and miR-501-3p in lung and bone marrow. Normalized intensities of miR-378a-3p and miR-501-3p levels in lung and bone marrow (BM) of IL-33 challenged (purple circles) and PBS control mice (blue circles) assessed by miRNA microarray. N =4 mice/group. r_P, Pearson correlation coefficient.

Supplementary Figure S2. miRNA abundance by normalized microarray intensity signal partly resembles miRNAs quantified using Nanostring technology. A: Bar graph demonstrating miRNA abundance in bone marrow (BM) of PBS control mice by microarray, and IL-33 exposed mice visualized in heatmap below (demonstrating rank from most highly expressed; 1). Venn diagram demonstrating unique and shared miRNAs detected by microarray and Nanostring technology among top 50 expressed **(B)** and all miRNA probes included in microarray and Nanostring assays **(C)**. N =4-6 mice/group. Bar graphs are shown with error bars demonstrating standard deviation.

Supplementary Figure S3. Significantly altered genes in the bone marrow of IL-33 exposed mice. mRNAs altered by IL-33 (P<0.05) in bone marrow assessed by Nanostring technology. N =6 mice/group. Unpaired t test two-tailed.
